# Metabolomic and transcriptomic analyses of peach leaves and fruits in response to pruning

**DOI:** 10.1186/s12864-024-10549-y

**Published:** 2024-07-03

**Authors:** Xiao-Fei Liu, Xiu-Hong An, Xin-Miao Li, He Zhang, Hong-Bo Cao, Hai-Jiang Chen, Yi Tian

**Affiliations:** 1 College of Horticulture, Hebei Agricultural University, Baoding, Hebei 071000 China; 2 National Engineering Research Center for Agriculture in Northern Mountainous Areas, Agricultural Technology Innovation Center in Mountainous Areas of Hebei Province, Hebei Agricultural University, Baoding, Hebei 071000 China

**Keywords:** Pruned peach trees, Coexpression network analysis, Auxin, Tryptophan and glutathione metabolism, Flavonoid biosynthesis

## Abstract

**Background:**

Pruning is an important cultivation management option that has important effects on peach yield and quality. However, the effects of pruning on the overall genetic and metabolic changes in peach leaves and fruits are poorly understood.

**Results:**

The transcriptomic and metabolomic profiles of leaves and fruits from trees subjected to pruning and unpruning treatments were measured. A total of 20,633 genes and 622 metabolites were detected. Compared with those in the control, 1,127 differentially expressed genes (DEGs) and 77 differentially expressed metabolites (DEMs) were identified in leaves from pruned and unpruned trees (pdLvsupdL), whereas 423 DEGs and 29 DEMs were identified in fruits from the pairwise comparison pdFvsupdF. The content of three auxin analogues was upregulated in the leaves of pruned trees, the content of all flavonoids detected in the leaves decreased, and the expression of almost all genes involved in the flavonoid biosynthesis pathway decreased. The phenolic acid and amino acid metabolites detected in fruits from pruned trees were downregulated, and all terpenoids were upregulated. The correlation analysis revealed that DEGs and DEMs in leaves were enriched in tryptophan metabolism, auxin signal transduction, and flavonoid biosynthesis. DEGs and DEMs in fruits were enriched in flavonoid and phenylpropanoid biosynthesis, as well as L-glutamic acid biosynthesis.

**Conclusions:**

Pruning has different effects on the leaves and fruits of peach trees, affecting mainly the secondary metabolism and hormone signalling pathways in leaves and amino acid biosynthesis in fruits.

**Supplementary Information:**

The online version contains supplementary material available at 10.1186/s12864-024-10549-y.

## Background

Peach is one of the most anticipated summer fruits [1]. It has been popular for thousands of years. China is a major peach producer, with ca. 15 million metric tons (Mt) produced per year [2]. Peaches can be consumed fresh, canned, pureed, or juiced, and peach fruits are rich in vitamins, sugar, and minerals [3]. The enhanced growth and yield of peach trees require a variety of cultivation management practices during tree growth, such as fertilization, irrigation, weed control, and pest control [4, 5]. Furthermore, the sustained productivity and profitability of peaches require the control of tree sizes and canopies within the appropriate range for adequate nutrition [6, 7]. Pruning can ensure the specific height and spread of the tree and reduce self-shading to increase the photosynthetic area of the canopy [8, 9]. It can also maintain an appropriate balance between yield and vegetative growth [10, 11]. Therefore, pruning is important for the production of fruit trees, such as apple trees [12], mango trees [13], and cocoa [14].

Tree pruning is usually performed in winter and summer. In winter, the trees are dormant, and thus winter pruning can easily eliminate redundant, damaged, or dead branches without stimulating the germination of new shoots or damaging the trees [11]. However, since winter pruning reduces a tree’s cold tolerance for approximately two weeks, pruning in extremely cold weather leads to reduced flower bud survival and branch injury [10]. Currently, winter pruning is usually performed before the start of spring growth. Maggs [15] reported that winter pruning mainly affects shoot growth, as well as fruit quality and yield. In fact, summer pruning mainly affects light penetration and carbohydrate supply, which are important for fruit growth [16–20]. Summer pruning is performed during the tree growing season, and unproductive shoots or water sprouts are removed, thereby affecting the water status of trees [21]. Conesa et al. [22] reported that fruit mass and fruit diameter did not differ significantly between young and mature trees subjected to winter and summer pruning treatments, and soluble solid contents in young trees were significantly higher following winter pruning than following summer pruning, without significant differences in mature trees.

Pruning is the removal of plant parts to achieve a certain purpose [9, 23]. Like other forms of plant biomass removal, such as physical or herbivore-associated injuries, pruning is also a stress treatment for peach trees. Plants can adopt different strategies to cope with the effects of stress. Zhang et al. [24] reported that tea plants increased their own growth capacity to resist environmental stress. Waadt et al. [25] reported that phytohormone synthesis rapidly increased when plants were under stress. Zhang et al. [24] reported that pruning enhanced the expression of genes involved in plant hormone signal transduction, carotenoid biosynthesis, fatty acid biosynthesis and other pathways. Secondary metabolite biosynthesis was also affected by stress. In *Lonicera japonica*, pruning decreased the contents of the phenolic compounds luteoloside and chlorogenic acid, especially at the first harvest, and the downregulation of the *CHI* (*chalcone isomerase*) and *HQT* (*hydroxycinnamoyl CoA quinate hydroxycinnamoyl transferase*) genes was the main reason for the decrease in the aforementioned substances, whereas the expression of *PAL* (*phenylalanine ammonia-lyase*), *C4H2* (*cinnamate 4-hydroxylase 2*), *4CL* (*4-coumaroyl-CoA ligase*), and *CHS2* (*chalcone synthase*) was upregulated after pruning [26]. Sun et al. [27] showed that the expression of the catechin biosynthesis-related genes *PAL*, *4CL*, *CHS*, and *DFR* (*dihydroflavonol 4-reductase*) decreased in pruned tea leaves, whereas the expression of *SCPL1A* (*serine carboxypeptidase-like acyltransferases 1 A*) and *LAR* (*leucoanthocyanidin reductase*) increased. In *Larix kaempferi*, pruning also changed the expression of age-related genes, such as *AGL2* (*AGAMOUS-Like 2*), *SOC1-1* (*Suppressor of Overexpression of Constans 1–1*), and *AP2-2* (*APETALA2-1*), to rejuvenate trees [28].

Although peach tree pruning has been performed for decades, most studies have focused on the effect of pruning on branching and yield [10, 29]. The underlying mechanisms of pruning-induced tree growth and yield are poorly understood. Furthermore, the metabolic physiology of peach leaves and fruits undergoing pruning treatment is poorly understood. Therefore, in this study, we determined the global transcriptomic and metabolomic profiles of peach leaves and fruits from trees with and without pruning treatment to identify key genes and metabolites involved in the response to pruning.

## Methods

### Plant materials and treatments

The golden-flesh peach variety ‘Huangjinguan’ was grown in the specimen garden of Hebei Agricultural University and grafted onto the wild peach rootstock *Prunus persica*. The identification of the ‘Huangjinguan’ cultivar was performed by the Shandong Provincial Forestry Species Validation and Approval Committee in 2006, with accession number Lu R-SV-PPE-002-2007. No special permission was necessary to collect such samples. The experiment trees were 5 years old with a spacing of 1.5 × 4 m. The trees were pruned manually in winter, and approximately 70% of the branches were cut off. The unpruned trees were used as controls. Except for pruning, all trees were cultivated under natural growing conditions with the same cultivation management practices, such as irrigation, fertilization, and pest control. The leaves used to analyse the transcriptome and metabolome were collected in July. Mature leaves in the middle of branches outside the crown were sampled from four directions, and five leaves were taken from each direction. Mature fruits from different directions around the crown were collected 90 days after flowering for further analysis. After the peel was removed, the peach flesh was cut into small pieces. The samples were immediately frozen in liquid nitrogen. Five trees were used as replicates, and all samples were analysed in three independent biological replicates.

### Physiological measurements

The new shoots from pruned and unpruned trees were surveyed in July. The numbers of bouquet shoots (< 5 cm), short shoots (5–15 cm), middle shoots (15–30 cm), and long shoots (> 30 cm) were surveyed. The total shoot growth was calculated as the sum of the lengths of the new shoots. The proportions of new shoots were analysed in five independent biological replicates. The mature leaves for determining length and width were sampled in the same manner as described above. The longest extension from the leaf apex to the base without the petiole was measured as the leaf length, and the longest extension perpendicular to the leaf length was measured as the leaf width [30, 31]. Twenty ripe fruits were weighed, and the average fruit weight was calculated. Fruit soluble solid levels were measured using a digital refractometer.

### Transcriptome analysis

Transcriptome sequencing was performed by MetWare Company (Wuhan, China). Total RNA was extracted from leaves and fruits using the CTAB method [32]. Transcriptome data were sequenced on the Illumina HiSeq platform [33]. Clean reads were assembled and aligned to the peach reference genome (https://www.rosaceae.org/species/prunus_persica/genome_v2.0.a1) using HISAT2 [34]. The fragments per kilobase of exon per million mapped reads (FPKM) value of each gene was calculated based on the length of the gene and the read count mapped to the gene. Differentially expressed genes (DEGs) were identified using |log2fold change|≥1 and FDR (False Discovery Rate) < 0.05 [35]. The functions of the DEGs were annotated using the Gene Ontology (GO) [36] and Kyoto Encyclopedia of Genes and Genomes (KEGG) databases (https://www.genome.jp/kegg) [37].

### Metabolomic analysis

The metabolomic analyses were performed by MetWare Company (Wuhan, China). The extraction, detection, and quantification of metabolites were performed following standard procedures [38–40]. The metabolites in leaves and fruits were extracted in 70% methanol at 4 °C overnight and then centrifuged at 12,000 rpm for 10 min. The extracts were analysed using a UPLC-MS/MS system. Separation was achieved on an Agilent SB-C18 column (2.1 mm × 100 mm, 1.8 μm) with a gradient elution system consisting of acetonitrile and water containing 0.1% formic acid. The flow rate was 0.35 ml/min, and the sample volume was 4 μl. All detected metabolites were annotated based on the MetWare self-built database. Metabolite quantification was performed using multiple reaction monitoring (MRM). The metabolite data were analysed using Analyst 1.6.3 software. Metabolites with a VIP ≥ 1 and a fold change ≥ 2 or ≤ 0.5 were considered differentially expressed metabolites (DEMs). The DEMs were mapped to KEGG pathways [37] to show differences in metabolite pathway enrichment.

### Coexpression network analysis of the metabolome and transcriptome

Pearson’s correlation coefficients were calculated based on the fold changes of each DEG and DEM. Correlations corresponding to each coefficient with R^2^ > 0.8 and PCCP < 0.05 were selected. Cytoscape (version 3.8.2) was used to map the relationships between metabolites and genes.

### Quantitative RT‒PCR validation of DEGs

qRT‒PCR was performed using mRNA obtained from peach leaves and fruits to validate the RNA-seq data. Twenty DEGs involved in flavonoid biosynthesis, amino acid metabolism, and hormone signal transduction were selected from the transcriptomes for validation. The method was performed as described previously [41]. The relative expression of genes was calculated using the 2^−ΔΔCT^ method. The primers used for qRT‒PCR are shown in Table S1.

### Statistical analysis

The data are shown as the means ± standard errors of independent biological replicates. The data were analysed with SPSS 20.0 and Excel 2016.

## Results

### Growth responses of peach trees subjected to pruning treatment

**Fig. 1:**
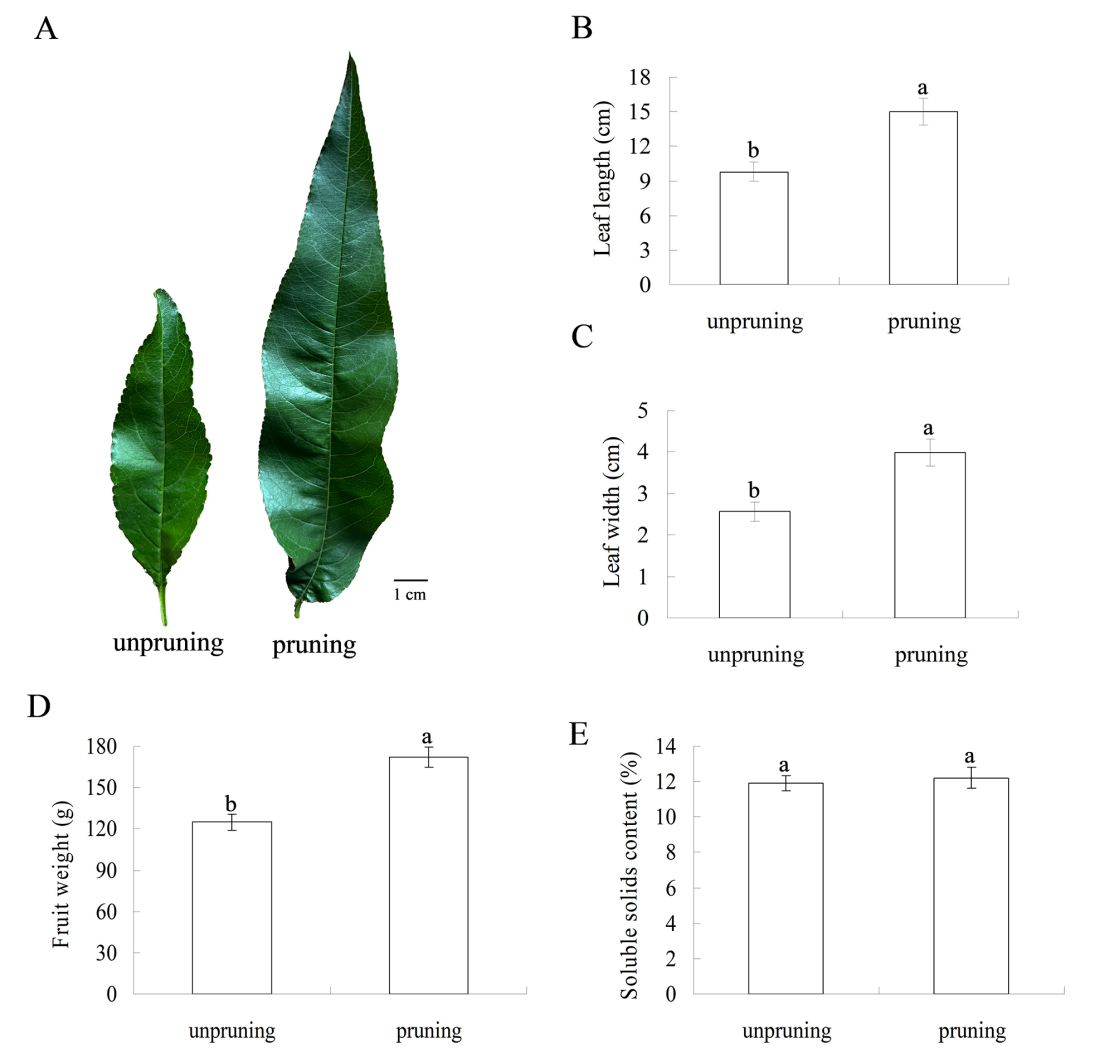
Phenotypic and physiological responses of peach trees subjected to pruning treatment. **A**, Phenotypes of leaves from pruned and unpruned peach trees. **B**-**C**, The values of leaf length and leaf width. **D**-**E**, Fruit weight and soluble solid content. The values are presented as the means ± SDs of three independent biological replicates, and different lowercase letters above the bars indicate significant differences among the different treatments (*P* < 0.05)

Approximately 96 new shoots were found on pruned trees, significantly fewer than the 226 observed on unpruned trees. However, the total new shoot length of the pruned trees was 1,177 cm, which was nearly twice that of the unpruned trees, and thus the average new shoot length was longer for the pruned trees. Unpruned trees were dominated by bouquets and short shoots, which were nearly 5-fold and 2-fold greater than those of pruned trees, respectively. In contrast, the pruned trees carried 30 middle shoots and 7 long shoots, which were almost absent in the unpruned trees (Table 1). In addition, we investigated whether pruning had any effect on leaves or fruits. The mature leaves from the pruned trees were larger than those from the unpruned trees (Fig. 1A). Both leaf length and leaf width were significantly greater in these plants than in unpruned plants (Fig. 1B, C). Moreover, the fruits from pruned trees grew larger than those from unpruned trees, and the fruit weight increased significantly compared with that of unpruned trees (Fig. 1D). However, no significant differences were observed between pruned and unpruned trees in terms of the soluble solid contents of fruits (Fig. 1E).

**Table 1:** The proportions of tree components in pruned and unpruned trees

Treatment	Total shoot length (cm)	Total number of shoots	Average shoot length (cm)	Number of bouquet branch	Number of short branch	Number of middle branch	Number of long branch
unpruning	632.6 ± 19.03	225.6 ± 11.39	2.8	182.8 ± 7.5	41 ± 3.08	2 ± 1.58	0
pruning	1177.4 ± 24.4	95.8 ± 4.207	12.29	37.2 ± 1.64	21.6 ± 2.07	30.4 ± 2.79	7 ± 1

### Transcriptome analysis of peach leaves and fruits undergoing pruning

A transcriptome analysis of leaves and fruits from pruned and unpruned trees was performed to comprehensively explore the effects of pruning on peach tree growth and development at the gene level. A total of 12 samples were sequenced, and 81.87 Gb of clean data were obtained. The percentages of Q30 bases ranged from 94.49 to 95.3%, and the percentages of GC content were between 45.42% and 45.8% (Table S2). A total of 20,633 genes were annotated in the databases (Table S3). A total of 1,127 DEGs were identified in the leaves of pruned and unpruned trees (pdLvsupdL), with 357 upregulated and 770 downregulated genes (Table S4). The fruits subjected to the pairwise comparison pdFvsupdF exhibited 423 DEGs, including 247 upregulated and 176 downregulated genes (Table S5). These results suggested that pruning affects the expression of genes in peach leaves and fruits.

### Functional analysis of DEGs in peach leaves and fruits following pruning treatment

**Fig. 2:**
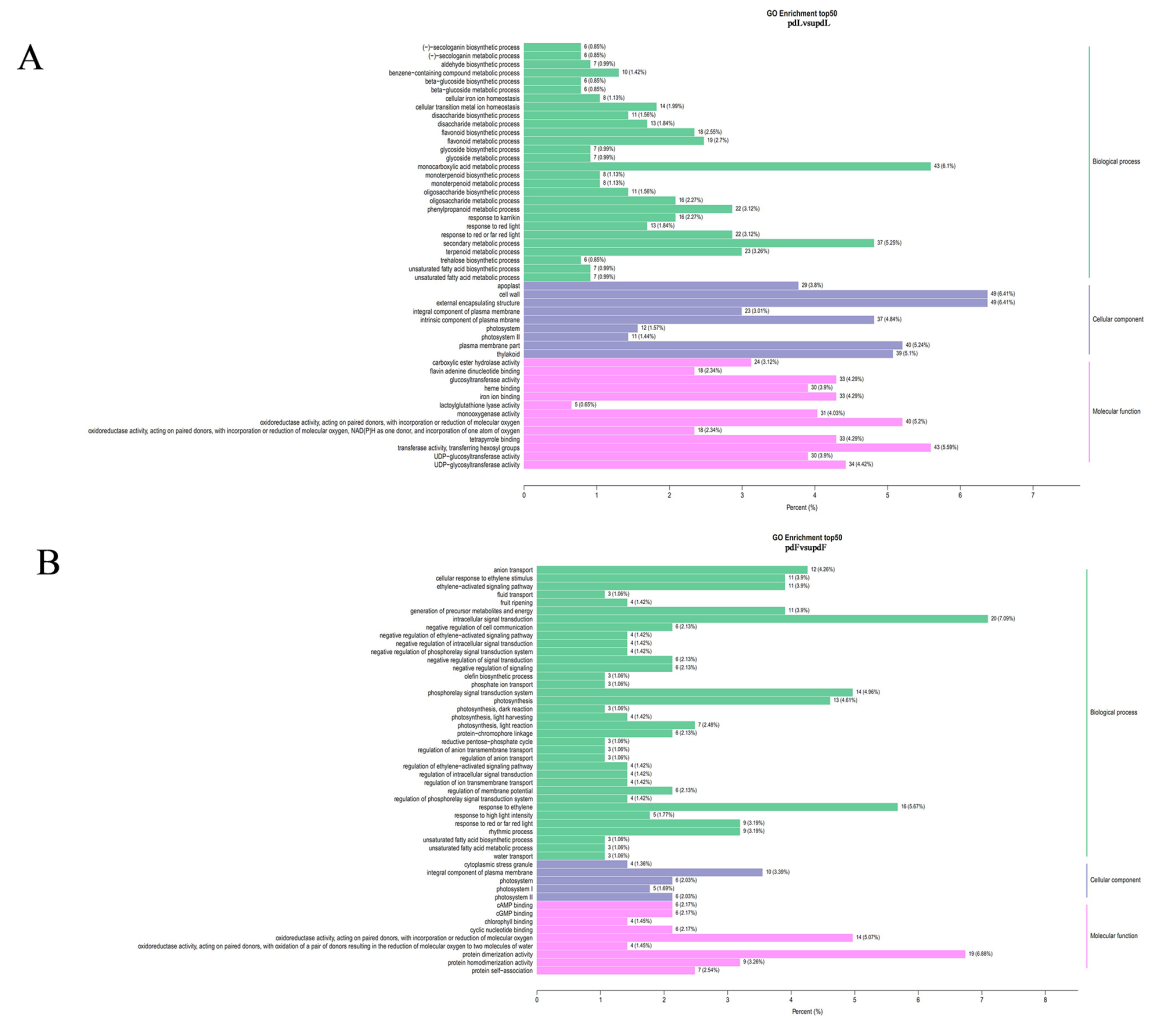
DEGs enriched in different GO terms. **A**, GO terms of DEGs in pdLvsupdL. **B**, GO terms of DEGs in pdFvsupdF. The Y-axis represents GO terms. The X-axis indicates the number of DEGs. All GO terms are grouped into three ontologies: green for biological processes, purple for cellular components and pink for molecular functions

GO functional enrichment analysis was conducted to understand the biological functions of the DEGs in peach leaves and fruits subjected to pruning treatment. All DEGs were classified into biological processes, cellular components, and molecular functions (Tables S6 and S7). However, the DEGs in pdLvsupdL and pdFvsupdF differed according to the GO enrichment analysis. In the biological process category, DEGs from pdLvsupdL were mainly enriched in monocarboxylic acid metabolic processes and secondary metabolic processes. In the cellular component category, DEGs were mainly enriched in the cell wall, external encapsulating structure, and plasma membrane. In the molecular function category, DEGs were mainly enriched in transferase activity and oxidoreductase activity (Fig. 2A). However, DEGs in pdFvsupdF were mainly enriched in intracellular signal transduction, response to ethylene, phosphorelay signal transduction, and photosynthesis in the biological process category (Fig. 2B). In addition, the DEGs in pdFvsupdF were also enriched in protein dimerization and oxidoreductase activities in the molecular function category.

**Fig. 3:**
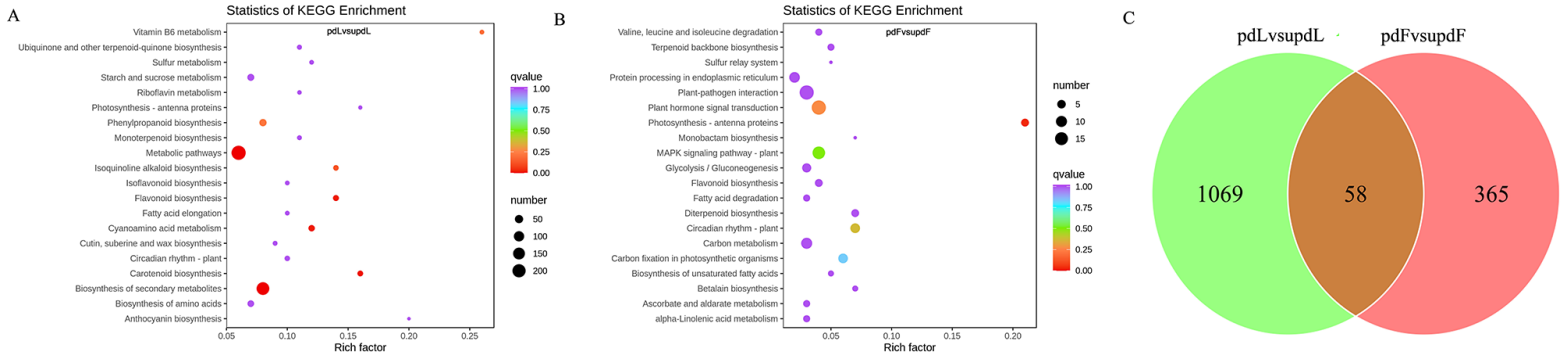
DEGs enriched in different KEGG pathways identified via a Venn analysis. **A**, KEGG pathway analysis of DEGs in pdLvsupdL. **B**, KEGG pathway analysis of DEGs in pdFvsupdF. **C**, Venn diagram of genes in pdLvsupdL and pdFvsupdF

Furthermore, all DEGs were distributed into 123 KEGG pathways, and more genes were distributed in metabolic pathways and biosynthesis of secondary metabolites (Tables S8 and S9). KEGG enrichment analysis revealed that the DEGs in pdLvsupdL were significantly enriched in starch and sucrose metabolism, phenylpropanoid biosynthesis, flavonoid biosynthesis, cyanoamino acid metabolism, carotenoid biosynthesis, biosynthesis of amino acids, and other categories (Fig. 3A). However, the DEGs in pdFvsupdF were enriched in plant hormone signal transduction, MAPK signalling in plants, carbon fixation in photosynthetic organisms, plant‒pathogen interaction, carbon metabolism, glycolysis/gluconeogenesis, and other categories (Fig. 3B).

The DEGs in pdLvsupdL and pdFvsupdF were visualized using a Venn diagram, and the expression of 58 genes, including *CHS*, *FLS* (*flavonol synthase*), *ARR* (*two-component response regulator*), *EIN3* (*ethylene-insensitive protein 3*), *GLB* (*beta-galactosidase*), and *P5CS* (*delta-1-pyrroline-5-carboxylate synthase*), varied in both leaves and fruits after pruning (Fig. 3C, Table S10), indicating that pruning may have different effects on genes in leaves and fruits.

### Metabolome analysis of peach leaves and fruits undergoing pruning

**Fig. 4:**
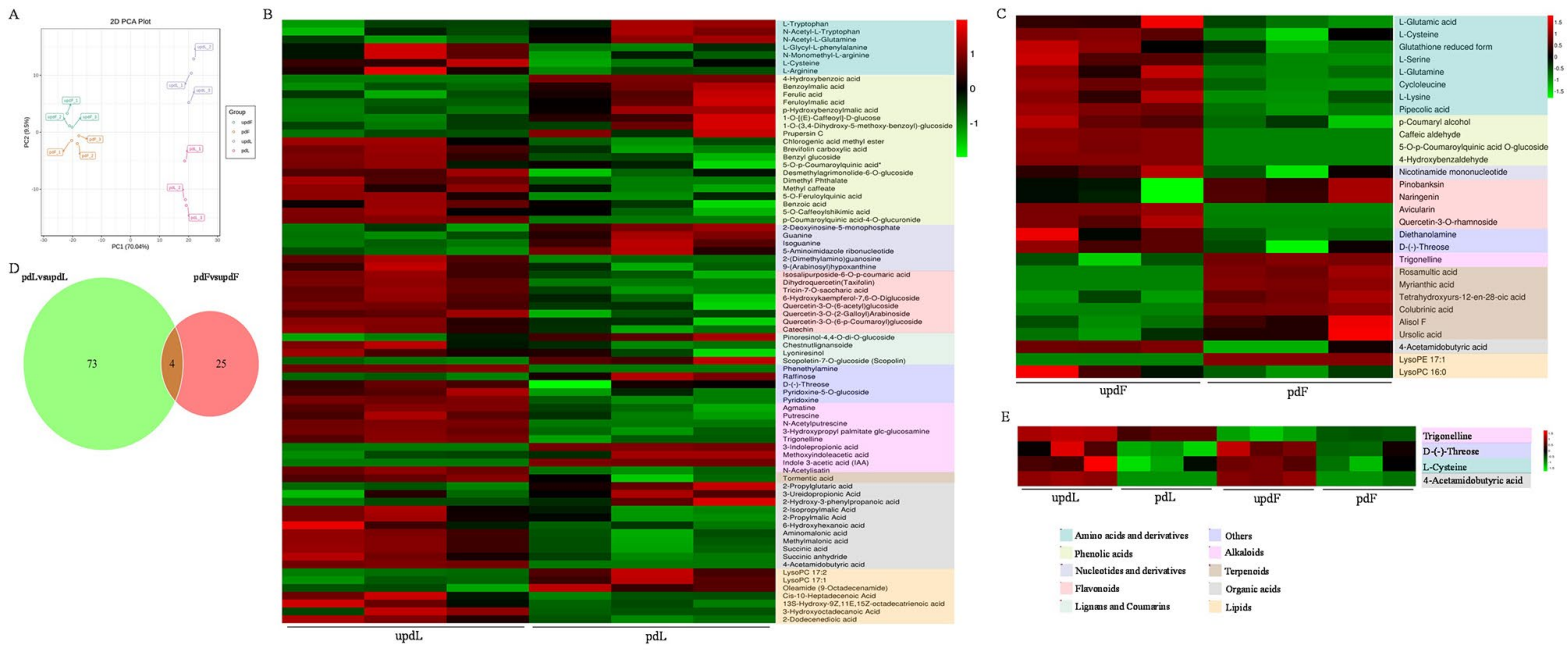
Metabolite analysis in peach trees undergoing pruning. **A**, PCA of metabolites. **B**, Metabolite analysis of pdLvsupdL. **C**, Metabolite analysis of pdFvsupdF. **D**-**E**, Venn analysis of metabolites in pdLvsupdL and pdFvsupdF

A metabolome analysis was performed to analyse the changes in global metabolites in peach leaves and fruits after pruning treatment. A total of 622 metabolites was detected (Table S11), which were divided into four groups according to PCA (Fig. 4A). Based on a |fold change| > 2 and VIP > 1, 77 and 29 DEMs were identified in pdLvsupdL and pdFvsupdF, respectively. A total of 27 upregulated and 50 downregulated metabolites, including flavonoids (8), alkaloids (9), phenolic acids (19), organic acids (11), and other compounds, were detected in pdLvsupdL (Fig. 4B, Table S12). All flavonoids, including catechin, tricin-7-O-saccharic acid, 6-hydroxykaempferol-7,6-O-diglucoside, quercetin-3-O-(6’’-acetyl) glucoside, quercetin-3-O-(2’’-galloyl) arabinoside, quercetin-3-O-(6’’-p-coumaroyl) glucoside, isosalipurposide-6’’-O-p-coumaric acid, and dihydroquercetin, were downregulated after pruning. Notably, 3 active auxin compounds, namely, indole 3-acetic acid, 3-indolepropionic acid, and methoxyindoleacetic acid, were upregulated in the leaves after pruning. In pdFvsupdF, 10 upregulated and 19 downregulated metabolites, including terpenoids (6), lipids (2), phenolic acids (4), flavonoids (4), amino acids and derivatives (8), were detected (Fig. 4C, Table S13). Among them, all phenolic acids (p-coumaryl alcohol, caffeic aldehyde, 5-O-p-coumaroylquinic acid O-glucoside, and 4-hydroxybenzaldehyde) and amino acid metabolites (L-glutamic acid, L-cysteine, glutathione reduced form, L-serine, L-glutamine, cycloleucine, L-lysine, and pipecolic acid) were downregulated, and all terpenoids (rosamultic acid, myrianthic acid, 1β,2α,3α,19α-tetrahydroxyurs-12-en-28-oic acid, colubrinic acid, alisol F, and ursolic acid) were upregulated.

A Venn diagram was used to show the relationships between the DEMs in pdLvsupdL and pdFvsupdF and to test whether pruning had the same effects on metabolites in leaves and fruits, and only 4 DEMs coexisted in these two groups (Fig. 4D). Trigonelline was upregulated in leaves and vice versa. However, D-(-)-threose, L-cysteine, and 4-acetamidobutyric acid were downregulated in the two groups (Fig. 4E). These results indicated that pruning had different effects on metabolites in peach leaves and fruits.

### Coexpression network analysis of DEGs and DEMs in peach leaves and fruits subjected to pruning treatment

**Fig. 5:**
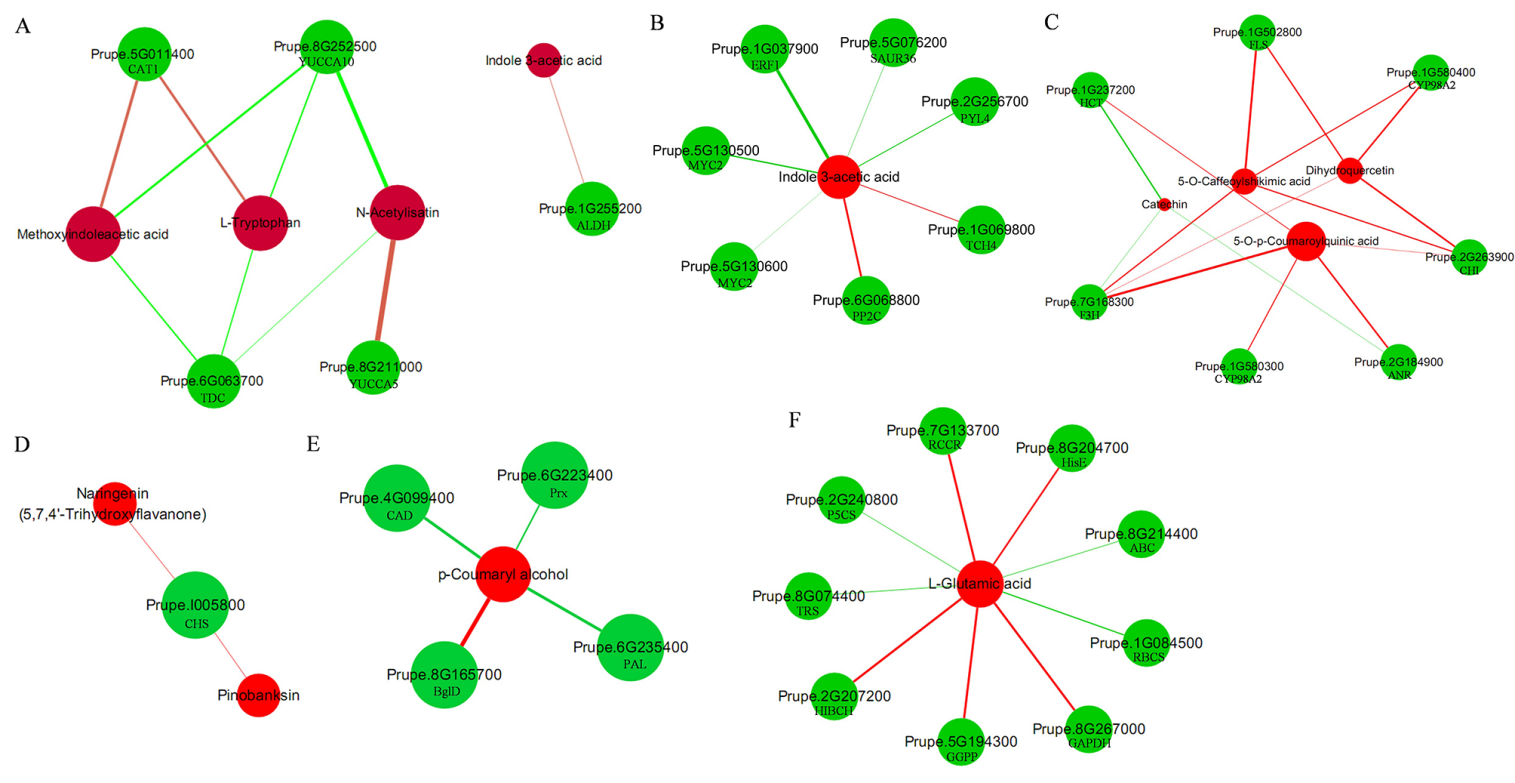
Coexpression analysis of DEGs and DEMs based on Pearson’s correlations (|Pearson’s correlation coefficient|> 0.8 and PCCP < 0.05). **A**-**C**, Interaction network of DEGs and DEMs involved in tryptophan metabolism, auxin signal transduction, and flavonoid biosynthesis in pdLvsupdL. **D**-**F**, Interaction network of DEGs and DEMs involved in phenylpropanoid biosynthesis, flavonoid biosynthesis, and L-glutamic acid metabolism in pdFvsupdF. Red circles represent metabolites, and green circles represent genes. Lines coloured in red and green represent positive and negative correlations, respectively. The thicker the lines, the stronger the correlation. The larger the red circle, the greater the number of genes involved. CAT1, catalase isozyme 1; YUCCA10, indole-3-pyruvate monooxygenase; TDC, L-tryptophan decarboxylase; YUCCA5, indole-3-pyruvate monooxygenase; ALDH, aldehyde dehydrogenase; SAUR36, auxin-responsive protein; TCH4, xyloglucan: xyloglucosyl transferase; PP2C, phosphatase 2 C; ERF1, ethylene-responsive transcription factor 1; MYC2, myelocytomatosis 2; PYL4, abscisic acid receptor; ANR, anthocyanidin reductase; HCT, vinorine synthase; F3H, naringenin 3-dioxygenase; CYP98A2, cytochrome P450 98A2; FLS, flavonol synthase; CHI, chalcone isomerase; BglD, beta-glucosidase; CAD, cinnamyl-alcohol dehydrogenase; Prx, peroxidase; PAL, phenylalanine ammonia-lyase; CHS, chalcone synthase; GAPDH, glyceraldehyde-3-phosphate dehydrogenase; ABC, ATP-binding cassette; HisE, phosphoribosyl-ATP pyrophosphohydrolase; TRS, threonyl-tRNA synthetase; RCCR, red chlorophyll catabolite reductase; GGPP, geranylgeranyl diphosphate; P5CS, delta-1-pyrroline-5-carboxylate synthetase; HIBCH, 3-hydroxyisobutyryl-CoA hydrolase; RBCS, ribulose-bisphosphate carboxylase small chain

A correlation analysis was conducted to determine the relationships between DEGs and DEMs in peach leaves and fruits subjected to pruning (|Pearson correlation coefficient|> 0.8 and PCCP < 0.05; Tables S14 and S15). In leaves, the coexpression networks of DEGs and DEMs were enriched in tryptophan metabolism (e.g., *CAT1*, *TDC*, *YUCCA*, *ALDH*, indole 3-acetic acid, L-tryptophan, and methoxyindoleacetic acid) (Fig. 5A), auxin signal transduction (e.g., *SAUR36*, *TCH4*, *PP2C*, and indole 3-acetic acid) (Fig. 5B), and flavonoid biosynthesis (e.g., *F3H*, *CHI*, *ANR*, *FLS*, catechin, dihydroquercetin, and 5-O-caffeoylshikimic acid) (Fig. 5C). However, except for flavonoid biosynthesis (*CHS*, pinobanksin, and naringenin) (Fig. 5D), the coexpression networks of DEGs and DEMs were enriched in phenylpropanoid biosynthesis (*CAD*, *Prx*, *PAL*, *BglD*, and p-coumaryl alcohol) (Fig. 5E) and L-glutamic acid metabolism (e.g., *ABC*, *GGPP*, and L-glutamic acid) (Fig. 5F) in fruits.

### Integrated analysis of genes and metabolites related to tryptophan metabolism in leaves subjected to pruning treatment

**Fig. 6:**
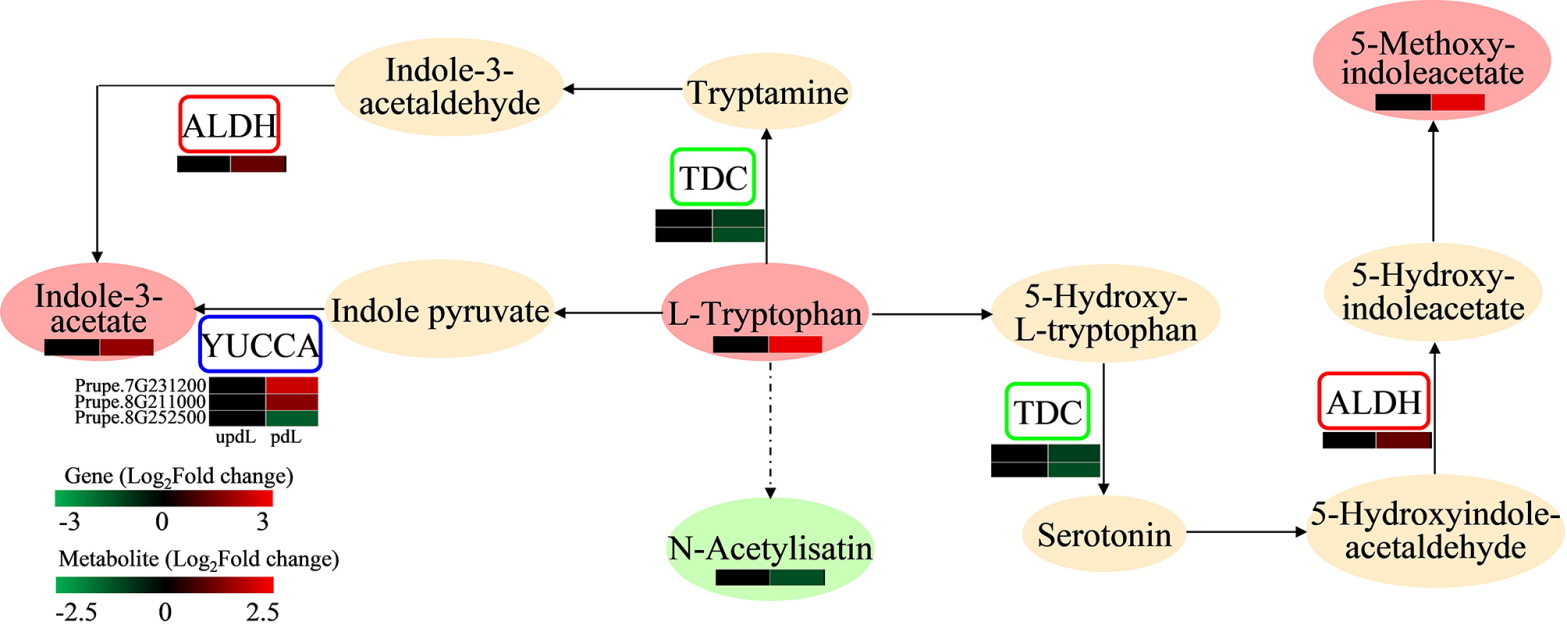
The DEGs and DEMs involved in tryptophan metabolism in peach leaves subjected to pruning (|fold change|> 1 and *p* value < 0.05). The ovals represent metabolites. The rectangles represent genes. The orange pattern represents metabolites that did not change following pruning treatment. The blue pattern represents genes whose expression changed following pruning treatment. The red pattern represents metabolites or genes that were upregulated following pruning treatment. The green pattern represents metabolites or genes that were downregulated after pruning treatment. The left part of the heatmap represents before pruning, and the right part represents after pruning. Red and green in the heatmap indicate up- and downregulation, respectively

The interactions between DEGs and DEMs related to tryptophan metabolism were analysed to assess the effects of pruning on genes and metabolites related to tryptophan metabolism in leaves (Fig. 6, Table S16). Six DEGs were found to be related to tryptophan metabolism in pdLvsupdL. Among these genes, 2 *TDC* (*tyrosine decarboxylase 1*, Prupe.6G063700 and Prupe.8G214500) genes and 1 *YUCCA* (*indole-3-pyruvate monooxygenase*, Prupe.8G252500) gene were downregulated. In addition, 1 *ALDH* (*aldehyde dehydrogenase*, Prupe.1G255200) gene and 2 *YUCCA* genes (Prupe.7G231200 and Prupe.8G211000) were upregulated. L-Tryptophan, methoxyindoleacetic acid, and indole 3-acetic acid (IAA) accumulated; however, N-acetylisatin levels decreased in pruned leaves. These results revealed that pruning activated the tryptophan metabolism pathway, and more active auxin substances accumulated in the leaves after pruning.

### Integrated analysis of genes and metabolites related to flavonoid biosynthesis in leaves subjected to pruning treatment

**Fig. 7:**
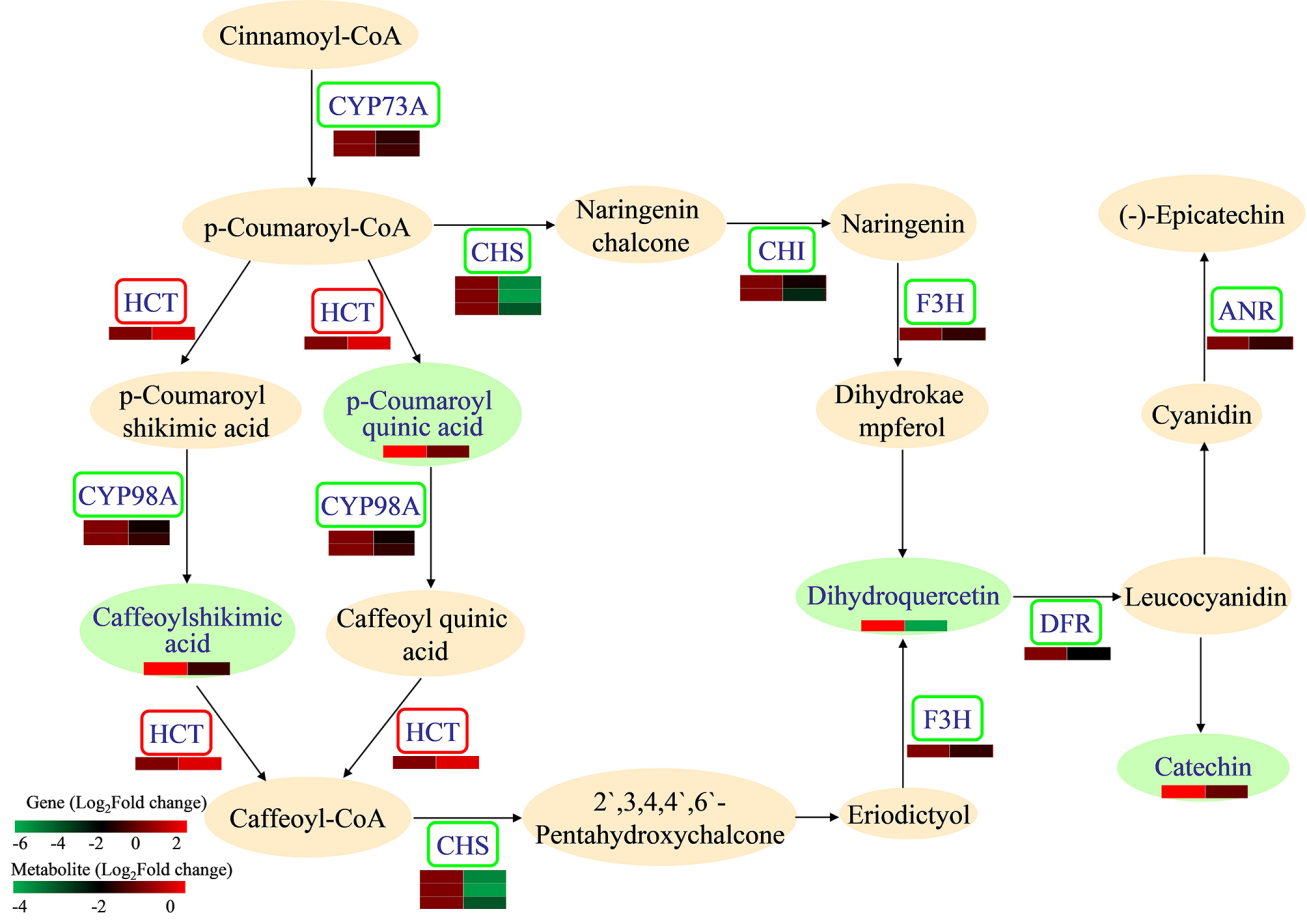
The DEGs and DEMs involved in flavonoid biosynthesis in peach leaves subjected to pruning (|fold change|> 1 and *p* value < 0.05). The ovals represent metabolites. The rectangles represent genes. The orange pattern represents metabolites that did not change after pruning treatment. The red pattern represents genes that were upregulated after pruning treatment. The green pattern represents metabolites or genes that were downregulated after pruning treatment. The left part of the heatmap represents before pruning, and the right part represents after pruning. Red and green in the heatmap indicate up- and downregulation, respectively

The interactions between DEGs and DEMs related to flavonoid biosynthesis were analysed to determine the effects of pruning on genes and metabolites related to flavonoid biosynthesis in leaves (Fig. 7, Table S17). *CYP73A* (*cytochrome P450*, Prupe.1G064900 and Prupe.6G040400), *CYP98A* (Prupe.1G580300 and Prupe.1G580400), *CHS* (Prupe.1G003000, Prupe.I005700, and Prupe.I005800), *CHI* (Prupe.2G225200 and Prupe.2G263900), *F3H* (*naringenin 3-dioxygenase*, Prupe.7G168300), *DFR* (*bifunctional dihydroflavonol 4-reductase*, Prupe.1G376400), and *ANR* (*anthocyanidin reductase*, Prupe.2G184900) were downregulated. However, *HCT* (*vinorine synthase*, Prupe.1G237200) was upregulated after pruning. Furthermore, the levels of metabolites related to flavonoid biosynthesis, including 5-O-p-coumaroylquinic acid, 5-O-caffeoylshikimic acid, dihydroquercetin, and catechin, were reduced in pruned leaves. These results indicated that pruning inhibited the flavonoid biosynthesis pathway in leaves.

### Integrated analysis of genes and metabolites related to glutathione metabolism in fruits obtained from pruned trees

**Fig. 8:**
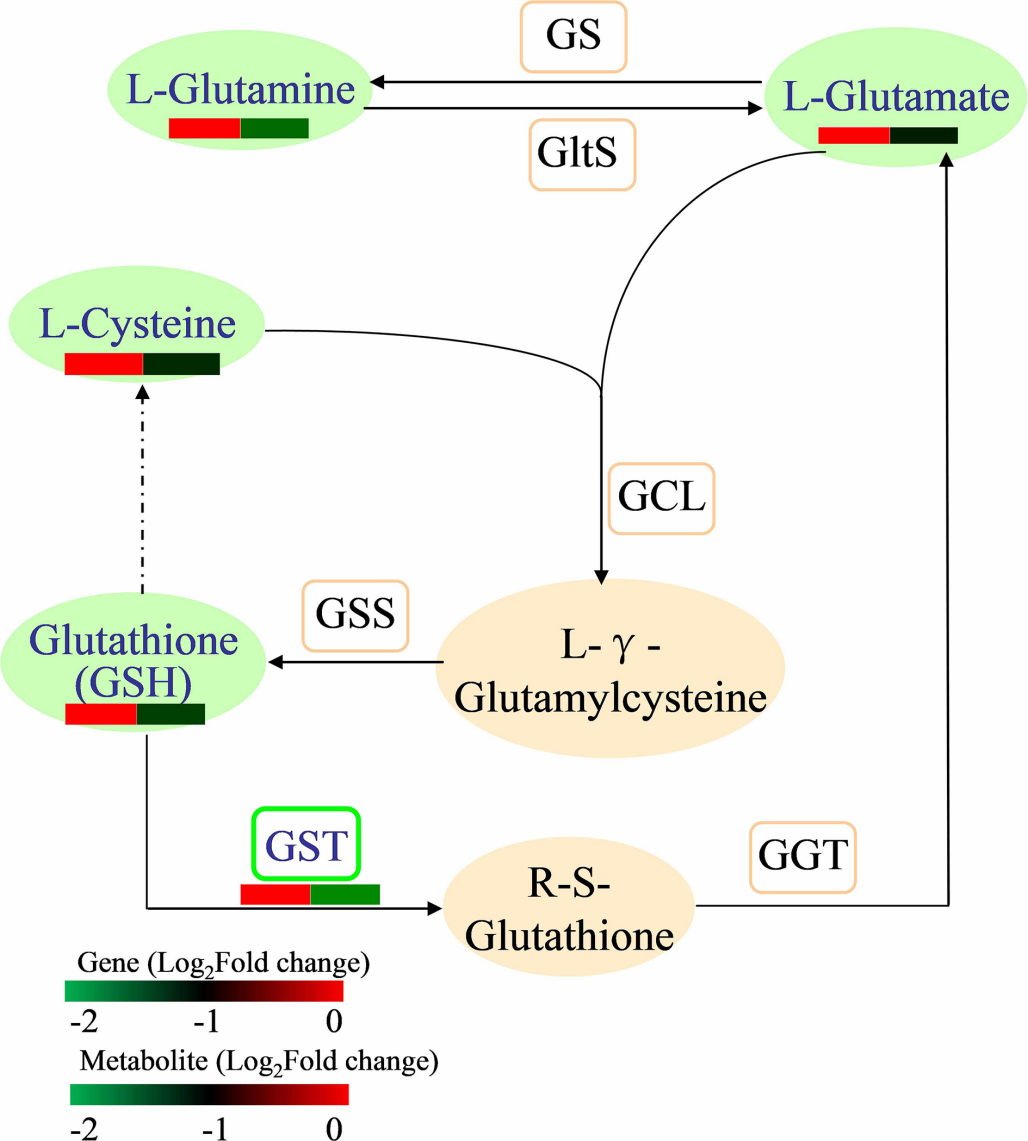
The DEGs and DEMs involved in glutathione metabolism in peach fruits subjected to pruning (|fold change|> 1 and *p* value < 0.05). The orange pattern represents the metabolites and genes that did not change after pruning treatment. The green pattern represents metabolites or genes that were downregulated after pruning treatment. Red and green in the heatmap indicate up- and downregulation, respectively

The interactions between DEGs and DEMs related to glutathione metabolism were analysed to test the effect of pruning on genes and metabolites related to glutathione metabolism in fruits (Fig. 8, Table S18). The *GST* (*glutathione S-transferase*, Prupe.6G040700) gene was downregulated. The levels of L-glutamine, L-glutamic acid, L-cysteine, and reduced glutathione (GSH) decreased in the fruits after pruning treatment. These results showed that the DEGs and DEMs related to glutathione metabolism in fruits were inhibited in response to pruning treatment.

### Expression of auxin-related genes after pruning treatment

**Fig. 9:**
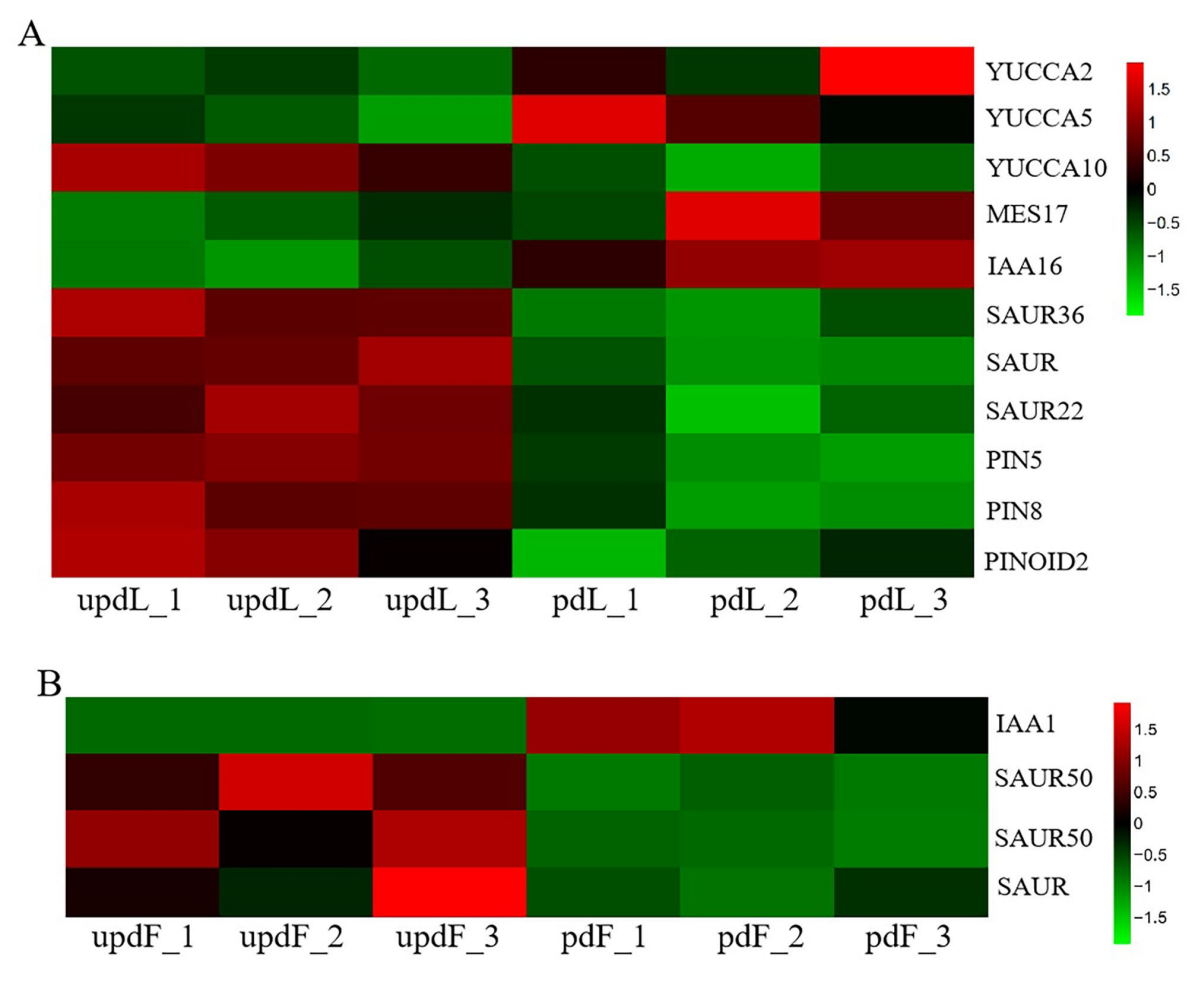
Expression of auxin-related genes in peach leaves (A) and fruits (B) undergoing pruning. The redder the colour, the greater the expression; the bluer the colour, the lower the expression

Pruning activated tryptophan metabolism and increased the accumulation of active auxin substances (indole 3-acetic acid, 3-indolepropionic acid, and methoxyindoleacetic acid) in leaves. Furthermore, the total shoot length, leaf size, and fruit weight increased. Therefore, we analysed auxin-related gene expression in leaves and fruits after pruning. In leaves, the expression of 3 *YUCCA* genes changed after pruning: *YUCCA2* (Prupe.7G231200) and *YUCCA5* (Prupe.8G211000) were upregulated, whereas *YUCCA10* (Prupe.8G252500) was downregulated. Members of the Aux/IAA family are early auxin response genes. After pruning, *IAA16* (Prupe.6G343800) was upregulated, but 3 SAUR genes, *SAUR* (Prupe.8G078700), *SAUR22* (Prupe.8G078800), and *SAUR36* (Prupe.5G076200), were downregulated in leaves. In addition, the auxin efflux carrier genes *PIN5* (Prupe.6G360300) and *PIN8* (Prupe.3G271700) and the protein kinase *PINOID2* (Prupe.4G088000) were also downregulated (Fig. 9A). In fruits, *IAA1* (Prupe.7G234800) was upregulated, and 3 *SAUR* genes (Prupe.6G108400, Prupe.7G167000, and Prupe.8G081100) were downregulated by pruning (Fig. 9B). These results indicated that pruning may regulate tree growth and development by altering auxin biosynthesis, transport, and signal transduction.

### qRT‒PCR validation of DEGs

qRT‒PCR was conducted to analyse the expression of 20 DEGs involved in flavonoid biosynthesis, amino acid metabolism, and hormone signal transduction from the transcriptome data and to test the accuracy of the DEGs obtained using RNA-seq. The qRT‒PCR results showed that all the detected gene expression trends were similar to those of the RNA-seq data (Fig. S1), indicating that the RNA-seq data were reliable.

## Discussion

Pruning is a principal cultivation management practice used to regulate tree structure and fruit yield [10]. Winter pruning is an irreplaceable strategy for improving the quality and yield of trees. Although pruning has been practised for many years in peach trees [10, 29], the effects of pruning on tree development have yet to be fully elucidated, especially the comprehensive effects of pruning on genes and metabolites in leaves and fruits. Therefore, we comprehensively analysed the variations in genes and metabolites in leaves and fruits obtained from pruned and unpruned peach trees to elucidate the response of peach trees to winter pruning.

Winter pruning stimulated the elongation of new shoots, and the proportions of tree components also changed. Bussi et al. [17] reported that the total number of young shoot lengths increased with increasing pruning intensity. Therefore, the average shoot length in pruned trees was longer than that in unpruned trees [23]. In our study, approximately 70% of the branches were cut off, implying heavy pruning. The total shoot length and average shoot length of the pruned trees were significantly longer than those of the unpruned trees (Table 1). Kumar et al. [3] reported that pruning treatments increased the average fruit weight of peach trees. The total soluble solid contents also increased after pruning, and especially in Saharanpur Prabhat peach, the soluble solid levels significantly increased with increasing pruning intensity. In our experiment, the average fruit weight also increased after pruning (Fig. 1D). However, the soluble solid content did not obviously change compared with that of unpruned trees (Fig. 1E). In addition, the leaves became larger after pruning (Fig. 1A). An increase in leaf area was also found in okra following a three-quarter pruning treatment [42]. Leaf expansion is thought to compensate for the reduction in leaf area caused by pruning, leading to an increase in dry weight [43].

For peach trees, pruning is also a stress treatment. Under stress, many secondary metabolic pathways are impacted in plants to improve their adaptability to the environment [24, 44]. Pruning affects metabolites in plants, but the changes in metabolites among different species, different varieties, or different tissues of the same variety are not consistent. Our study showed that more DEGs were enriched in biological processes in leaves and fruits (Fig. 2). In particular, in the leaves, DEGs were enriched in a series of biosynthetic and metabolic processes (Figs. 2A and 3A). Sun et al. [27] reported that DEGs associated with pruning were significantly enriched in the flavonoid pathway in tea leaves, and genes upstream of the catechin biosynthetic pathway were downregulated after pruning. Zhang et al. [24] also reported that the DEGs involved in flavonoid biosynthesis were all significantly downregulated after pruning. Rubel Mozumder et al. [45] reported that compared with pruned tea plants, the tea leaves from unpruned tea plants contained higher levels of caffeine, epicatechin, and gallocatechin and lower levels of γ-aminobutyrate, glutamate, and valine. In our study, pruning changed the contents of phenolic acids, flavonols, organic acids, amino acids and their derivatives in peach leaves (Fig. 4A). Coexpression network analysis revealed that the DEGs and DEMs from the leaves were enriched in flavonoid biosynthesis (Fig. 5C), and all the DEGs (except *HCT*) and DEMs in the flavonoid biosynthesis pathway were downregulated (Fig. 7). Perin et al. [46] reported that pruning had significant effects on must and wine quality-related metabolites. Pruning decreased the amino acid content in grape berries; however, the changes in the anthocyanin content differed among cultivars [46]. In our experiment, pruning changed the metabolite levels in fruits, with a decrease in terpenoids and increases in phenolic acids, amino acids and their derivatives (Fig. 4), indicating that pruning may affect the quality of peach fruits. DEGs and DEMs in the glutathione metabolism pathway were also downregulated in the fruits (Fig. 8). The flavonoid biosynthesis pathway and glutathione metabolism are involved in the stress response in plants [47, 48], and these changes may be a response to pruning to improve plant adaptability to the environment.

Pruning is thought to alter hormonal patterns in fruit trees by removing large areas of meristems, which are the source and sink of hormones [10]. Compared with unpruned trees, additional auxins, cytokinins (CKs), and gibberellins (GAs) were found in the aerial portions of trees under dormant pruning treatment [23]. Auxins play important roles in regulating plant growth, such as cell division and elongation, apical dominance, and blooming [49]. Auxins can be synthesized through the tryptophan pathway [50]. After pruning, more tryptophan accumulated in the leaves, and the content of active auxin compounds also increased (Figs. 4 and 6). ALDH and the YUC family are key enzymes that catalyse the conversion of indole-3-acetylaldehyde and indole-3-pyruvic acid into IAA, respectively. In addition, ALDH converts 5-hydroxyindole-acetaldehyde to 5-hydroxy-indoleacetate, which is the precursor of 5-methoxy-indoleacetate [49]. After pruning, the *ALDH* gene and 2 *YUCCA* genes were upregulated (Figs. 6 and 9), and the *ALDH* gene was positively correlated with IAA (Fig. 5A). The results indicated that pruning activated the tryptophan pathway, thus promoting the synthesis of auxins, which may be the main cause of the large leaves in pruned trees. Although we did not detect changes in the expression of other hormone metabolites, the expression of genes involved in CK, GA, ABA, jasmonic acid (JA), and ethylene signalling also changed after pruning treatment (Tables S4 and S5), and the expression of several genes related to ABA, JA, and ethylene signalling pathways showed positive and negative correlations with the expression of indole 3-acetic acid (Fig. 5B). Verma et al. [51] suggested that the signalling network and crosstalk between IAA, CKs, GA3, JA, and ABA modulate the plant defence response. Zhang et al. [24] also reported that pruning enhanced the expression of genes involved in plant hormone signal transduction. Pruning may affect various hormone signal transduction pathways, and these signalling pathways crosstalk with each other to regulate tree development and growth to improve plant adaptability to the environment.

## Conclusions

Winter pruning changed the proportions of tree components and increases the sizes of leaves and fruits. Winter pruning activated the tryptophan metabolism pathway and promoted active auxin substance accumulation in leaves. In addition, winter pruning inhibited the flavonoid biosynthesis pathway in leaves and the glutathione metabolism pathway in fruits. Secondary metabolism and auxin signalling pathways may be the main response to pruning to improve peach tree growth and developmental adaptability to the environment. Further investigation is needed to determine whether various hormone signalling networks and their crosstalk improve tree adaptability to the environment in response to pruning.

### Electronic supplementary material

Below is the link to the electronic supplementary material.

**Supplementary Material 1: Supplemental Table S1.** The primers used for qRT-PCR

**Supplementary Material 2: Supplemental Table S2.** Sequencing data statistics

**Supplementary Material 3: Supplemental Table S3.** The annotation of total reads

**Supplementary Material 4: Supplemental Table S4.** DEGs in the leaves undergoing pruning

**Supplementary Material 5: Supplemental Table S5.** DEGs in the fruits undergoing pruning

**Supplementary Material 6: Supplemental Table S6.** The GO terms enriched by the DEGs in leaves

**Supplementary Material 7: Supplemental Table S7.** The GO terms enriched by the DEGs in fruits

**Supplementary Material 8: Supplemental Table S8.** The KEGG enriched pathway by the DEGs in leaves

**Supplementary Material 9: Supplemental Table S9.** The KEGG enriched pathway by the DEGs in fruits

**Supplementary Material 10: Supplemental Table S10.** The venn analysis of DEGs in pdLvsupdL and pdFvsupdF

**Supplementary Material 11: Supplemental Table S11.** The information of metabolomes

**Supplementary Material 12: Supplemental Table S12.** DEMs in pdLvsupdL

**Supplementary Material 13: Supplemental Table S13.** DEMs in pdFvsupdF

**Supplementary Material 14: Supplemental Table S14.** Correlation analysis of DEGs and DEMs in leaves

**Supplementary Material 15: Supplemental Table S15.** Correlation analysis of DEGs and DEMs in fruits

**Supplementary Material 16: Supplemental Table S16.** The information of DEGs and DEMs involved in tryptophan metabolism in peach leaves

**Supplementary Material 17: Supplemental Table S17.** The information of DEGs and DEMs involved in flavonoid biosynthesis in peach leaves

**Supplementary Material 18: Supplemental Table S18.** The information of DEGs and DEMs involved in glutathione metabolism in peach fruits

**Supplementary Material 19: Supplemental Figure S1.** The qRT-PCR validation of selected genes

## Data Availability

Sequence data that support the findings of this study have been deposited in NCBI with the accession code PRJNA990955. The SRA submission will be released on 2024-06-01 or upon publication (https://submit.ncbi.nlm.nih.gov/subs/sra/SUB13605447/overview).

## Abbreviations

PCA: Principal Component Analysis
PCCP: Pearson Correlation Coefficient pvalue
VIP: Variable Importance in the Projection
