# Supplementary material for: Metabolomic and transcriptomic analyses of peach leaves and fruits in response to pruning
Source: BMC Genomics. 2024 Jul 3;25:666. doi: 10.1186/s12864-024-10549-y (PMC11223333; doi:10.1186/s12864-024-10549-y)
Supplement: Supplementary file 1 — Supplementary Material 1: Supplemental Table S1. The primers used for qRT-PCR [file 12864_2024_10549_MOESM1_ESM.doc]

Supplementary Table S1 The primers used for qRT-PCR

| ID | Gene name | Forward primer | Reverse primer |
| --- | --- | --- | --- |
| Prupe.1G003000 | CHS | CCAGCGCATGTGTAAGTACC | GTTTCACTCCATTTGTAGACAG |
| Prupe.1G502800 | FLS | GCTCATCTTCTCAGCCCTTAG | CGGTCGTGATCCCAGGTTG |
| Prupe.6G235400 | PAL | ACGCTGGAGTGTTTGGGAG | GTTGTTGAGGAACTTGGTG |
| Prupe.7G168300 | F3H | CAAATTCCTTTTCTCTCCAAC | GGCGCTCATCTTCGTCCCG |
| Prupe.2G225200 | CHI | CTCCCAATTTAAACGCACCG | CAGCCCCCTCACCCCTGCG |
| Prupe.6G343800 | IAA16 | CTACAGCCCATTCTAAACCAGC | GCTATTCCACCAGGCAACCCC |
| Prupe.2G005500 | LOX2.1 | CAGAGTTGGAGCAGGTGTTG | CATATAATAAATAGTCTAGTCCC |
| Prupe.6G054800 | ASNS | GAAGTGCATCAACAAGCTT | CACTAAGGCATCGCAAAGTCTC |
| Prupe.3G004100 | Lhcb1 | CATATTCTGCATACTCAATCATC | GAAACTCTACCATTGCCTTG |
| Prupe.8G148500 | SCPL17 | CAGTACATGCGCTTAGATTGC | CGTACCCAGTTTCAAGCTTG |
| Prupe.1G042500 | EIN3 | CCTCTCCCATGTCTCTCTA | GTTTCTTGAGTTGCCAGTAG |
| Prupe.7G167000 | SAUR50 | CTATATTCAAAGCTAGCAAG | GTTGTAACCATTTTTCTTCCC |
| Prupe.8G018700 | SAD | CTGAGTGAGGAAGAGCGAG | GGAATTTGGTGGAGTGAGGC |
| Prupe.1G023000 | ATPS | CCCTAAATTAAATCTCCTAC | CTTGGAACCGAGAGGGAAT |
| Prupe.6G182400 | TPPJ | CCACACCAGCACCTCAGTC | CCACGGGAGGCTTTTCGG |
| Prupe.1G034300 | ETR2 | CCTGCATGTGCTATAAGG | GTCACAATTGCACCGCGG |
| Prupe.8G249800 | GID1B | CACATCCTTTGCCAAACTC | CAGGAGATTGTAAGCTAGCTTG |
| Prupe.4G150200 | GA2ox | CTATCATCCATATCATCTTTCAGC | CTATGAGCTGTTTGGAGTCTGG |
| Prupe.7G244300 | EBF1 | CTTGAGATGTGGATGCTTGTAG | CGAACGCAAATTGGGAATC |
| Prupe.7G234800 | IAA1 | CACCCAACACCAAGAAGC | TAAGGTGAGCTCAGTGTCC |
