# Supplementary figures and images for: Metabolomic and transcriptomic analyses of peach leaves and fruits in response to pruning
Source: BMC Genomics. 2024 Jul 3;25:666. doi: 10.1186/s12864-024-10549-y (PMC11223333; doi:10.1186/s12864-024-10549-y)

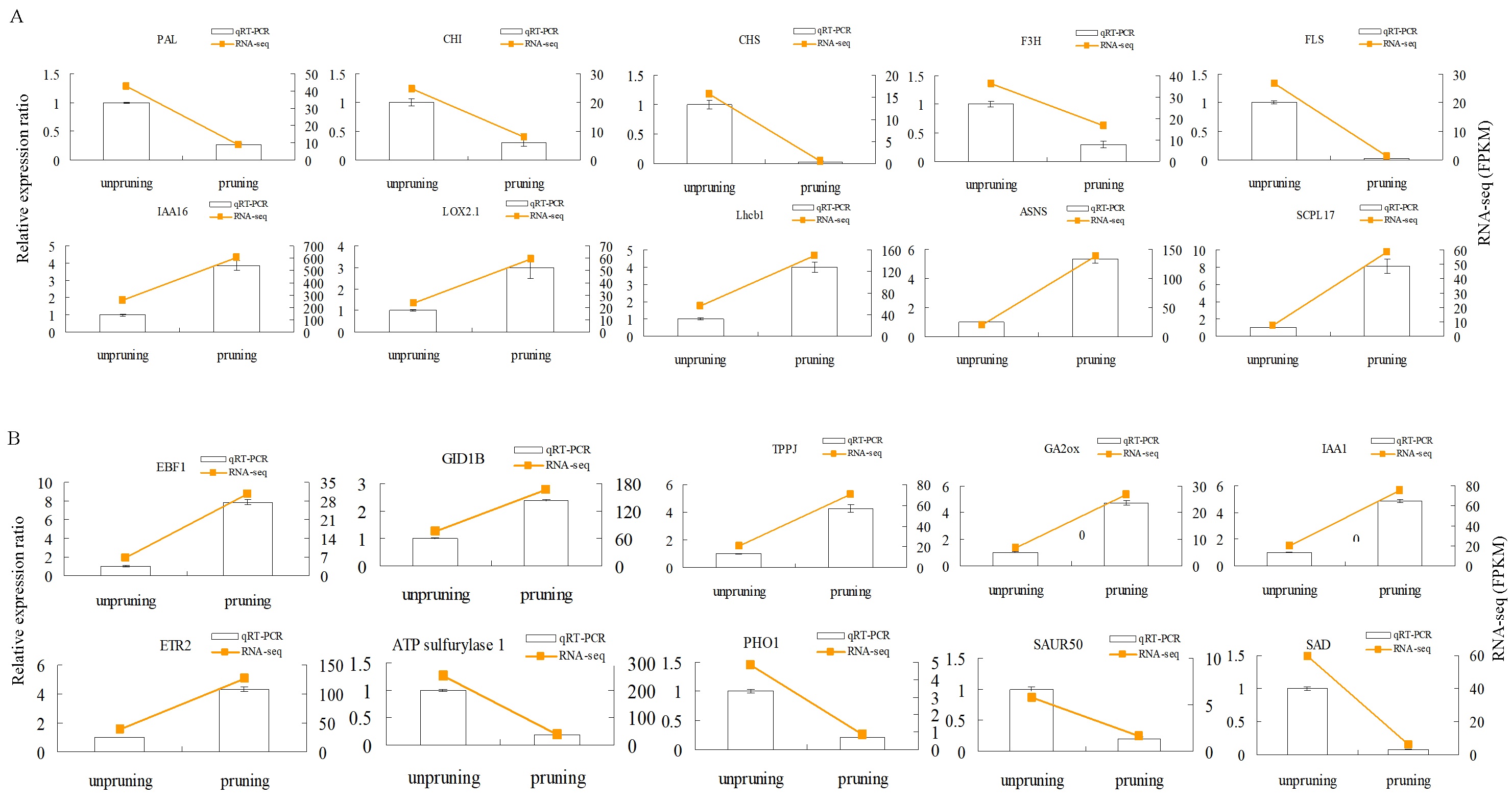

Supplement: Supplementary file 19 — Supplementary Material 19: Supplemental Figure S1. The qRT-PCR validation of selected genes [file 12864_2024_10549_MOESM19_ESM.jpg]
